# Generalized Erdős numbers for network analysis

**DOI:** 10.1098/rsos.172281

**Published:** 2018-08-29

**Authors:** Greg Morrison, Levi H. Dudte, L. Mahadevan

**Affiliations:** 1 Department of Physics, University of Houston, Houston, TX 77204, USA; 2 School of Engineering and Applied Sciences, Harvard University, Cambridge, MA 02138, USA; 3 Department of Physics, Harvard University, Cambridge, MA 02138, USA; 4 Department of Organismic and Evolutionary Biology, Harvard University, Cambridge, MA 02138, USA; 5 Kavli Institute for Nano-bio Science and Technology, Harvard University, Cambridge, MA 02138, USA

**Keywords:** network science, centrality, epidemic spreading

## Abstract

The identification of relationships in complex networks is critical in a variety of scientific contexts. This includes the identification of globally central nodes and analysing the importance of pairwise relationships between nodes. In this paper, we consider the concept of topological proximity (or ‘closeness’) between nodes in a weighted network using the generalized Erdős numbers (GENs). This measure satisfies a number of desirable properties for networks with nodes that share a finite resource. These include: (i) real-valuedness, (ii) non-locality and (iii) asymmetry. We show that they can be used to define a personalized measure of the importance of nodes in a network with a natural interpretation that leads to new methods to measure centrality. We show that the square of the leading eigenvector of an importance matrix defined using the GENs is strongly correlated with well-known measures such as PageRank, and define a personalized measure of centrality that is also well correlated with other existing measures. The utility of this measure of topological proximity is demonstrated by showing the asymmetries in both the dynamics of random walks and the mean infection time in epidemic spreading are better predicted by the topological definition of closeness provided by the GENs than they are by other measures.

## 1. Introduction

The study of complex networks has increased enormously in recent years due to their applicability to a wide range of physical [1,2], biological [3], epidemiological [4,5] and sociological [6] systems. Two basic goals in this regard are to understand and quantify the structure of the network to better characterize the relationship between the interacting members of the network (the nodes), while also characterizing the dynamical processes on the network [6] that may shed light on the processes by which they form [7].

Understanding the topological properties of the network on both a global and local level can be useful in approaching both of these goals. Global properties of interest may include simple measures of the distribution of node properties, such as the degree distribution, strength distribution or distribution of clustering coefficients [8,9]. Community structure in the network [10–12], which partitions the network into densely connected sub-networks with more links within communities than between communities, has been extensively studied and may provide more detailed information about the relationship between nodes than simple distributions. Community structure can indicate the existence of underlying similarities between nodes in the network, and may have a great impact on dynamical processes occurring on the network (such as a random walk [13–15] or epidemic spreading [4,16,17]), and can influence the material properties of granular systems [1].

While global properties of networks can be used to assess the attributes of the nodes on an aggregate level, it is also of great interest to understand the topological properties of nodes on an individual, local level. Node centrality is the classic example of a topological measure associated with an individual node, which assesses the ‘importance’ of a node in a variety of contexts. The most basic measure of a node's centrality is simply related to its degree, a property of the node that is based solely on the local topology of its connectivity. The centrality of individual nodes can also be measured incorporating the global topology of the network in a variety of ways, including PageRank [18], betweenness [15] or random walk [13] centralities. Each of these measures reduces the global properties of the network into an individualized local measure of importance, permitting a rank-ordering of their importance in the network [19,20]. Dynamics on networks can likewise be described in terms of pairwise interactions between nodes, with the time between an origin and a destination node (e.g. sources and sinks in a random walk or the time of infection of one node given an epidemic originating at another) depending on the network topology.

In many contexts [21,22], not all members of the network will necessarily agree on the importance of the same node: nodes that have a direct connection between them will be more important to each other than distant nodes in the network. Nodes that are central to the network as a whole may have very low importance from the perspective of sub-networks. The universality of importance is further complicated by the fact that we may expect the influence between a pair of nodes to be asymmetric even if they are directly connected [22] (the importance assigned by an important node towards an unimportant one is not necessarily the same as the importance assigned in the opposite direction), which may have important consequences in real-world systems [3]. The determination of a personalized measure of node importance that incorporates the global topology in an asymmetric measure is therefore an important but non-trivial problem.

In this paper, we explore the use of the generalized Erdős numbers [11,23] (GENs) as a measure of topological closeness between nodes in a network. Using the GENs, we identify two measures of centrality using the pairwise importance between nodes, and show that these global centralities are highly correlated with other common centrality measures. We show that the infection times of a node originating from a source that is not a nearest neighbour in an epidemic spreading model are highly correlated with the GENs, indicating their potential utility in predicting the influence of network topology on the dynamics on networks. We further show that the infection times are better predicted by the GENs than two other commonly used measures of the non-metric distance between nodes in a network: the resistance distance and mean first passage times (MFPT) in a random walk. Finally, we show that the asymmetry in the GENs is correlated with that in the MFPT between nodes in a random walk. This work illustrates that the GENs are a useful measure of the topological closeness between pairs of nodes in a complex network, and also illustrates that a meaningful definition of closeness has the potential to bridge the gap between the topology of a network and the dynamics on the network in multiple contexts.

## 2. The generalized Erdős numbers

### 2.1. Topological closeness in complex networks

When nodes represent objects in a physical space [2,24–27], the distance between nodes, *D*_*ij*_, is a naturally defined (metric) measure of closeness between the objects. Objects that are physically proximate (or close to one another) of course have small *D*_*ij*_ which is bounded below by *D*_*ij*_ = 0, while objects that are not close have large *D*_*ij*_. Owing to the generality of networks (where nodes and edges abstractly represent ‘objects’ and ‘interactions’, respectively), there can be no guarantee of a naturally defined distance metric [2,28], and, in some cases, the network topology itself must define a measure of closeness (Δ_*ij*_) based solely on the matrix of weights between nodes *i* and *j*, *w*_*ij*_ (with an undirected network where *w*_*ij*_ = *w*_*ji*_ is assumed throughout this paper).

The proximity or closeness between nodes, Δ_*ij*_, will be small for nodes that are close to one another and large for distant nodes, with a simple and common choice being Δ_*ij*_ = *w*^−1^_*ij*_ (so strongly connected nodes are ‘close’, and disconnected nodes are ‘far’). Alternatively, in an unweighted network, the length of the shortest path between a pair of nodes is a natural definition [28,29] and is the basis for the classic Erdős numbers in the context of an unweighted collaboration network [30].

**Figure 1.**
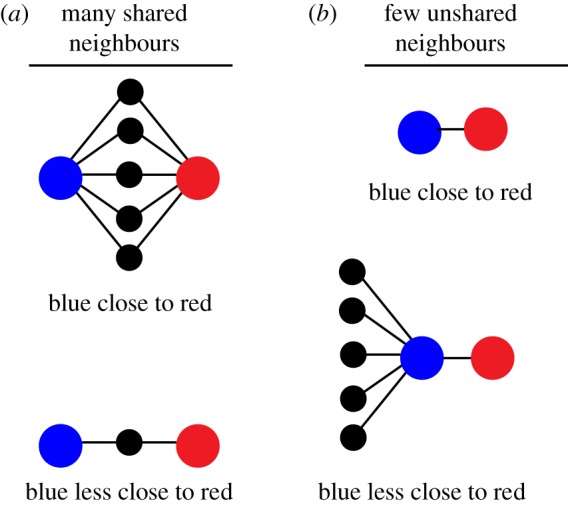
Two competing requirements for global ‘closeness’ in a network with shared resources. In (*a*), many short paths between nodes increase the closeness between them. This is similar to the resistance distance between nodes: additional parallel paths between them reduce their resistance distance. In (*b*), the finite resources of the high-degree blue node suggest that it should be less close to the red node than for the lower-degree blue node above, as resources are shared also with the other neighbours. This is similar to the transition probability from the blue node in a random walk: the more connections the blue node has, the lower probability of visiting the red node.

Improvements on this simple measure which incorporate the effect of multiple paths between nodes (see figure 1*a* for a schematic diagram) include the resistance distance [14,31], self-consistent similarity measures [32] and communicability [33], to name only a few. An additional approach to defining similarity between nodes is found by positing a multidimensional ‘latent space’ of node properties [34], with the assumption that nodes that are close in the latent space are likely to be connected in the network and each node's position in the space inferred from the observed connectivity. Each of these methods incorporates the global topology of the network into a symmetric measure of closeness between pairs of nodes (Δ_*ij*_ = Δ_*ji*_).

### 2.2. Finite resources and asymmetric measures of proximity

Finite resources are shared in some networks, with examples including collaboration on networks (where time with one collaborator reduces the available time for others), multi-core processor components [35] (where finite memory or other hardware must be shared) and random walks (where the walker can only move to a single neighbour at a time with a transition probability *P*_*i* → *j*_ = *w*_*ij*_/*W*_*i*_ with $W_{i}=\sum_{k}w_{ik}$ the total strength of the node *i*). In the context of these networks of limited resources, closeness measures such as resistance distance may be undesirable [22], because the addition of a new edge in the network should be detrimental to some nodes (those who receive less of the finite resource due to the new edge) and beneficial to others (those who receive more due to the edge). For closeness measures based on the direct weight between nodes (where the ‘closeness’ between *i* and *j* is often taken to be *w*^−1^_*ij*_) or resistance distance between nodes, it is straightforward to see that the newly measured closeness between nodes *i* and *j*, $\Delta_{ij}^{\left(\mathrm{new}\right)}\leq \Delta_{ij}^{\left(\mathrm{old}\right)}$ for all pairs, i.e. the addition of an edge can never cause nodes to become less close to one another. This is not sensible in the context of nodes that share a finite resource with their neighbours, as shown in figure 1*b*: if a node *i* has many neighbours, each receives less of the resource than if *i* had few neighbours.

The expectation of the influence of resource shared in figure 1 is satisfied by a number of existing measures of proximity. A quantity such as the transition probability in a random walk, *P*_*i* → *j*_, is asymmetric and ensures that nodes are closer if they have few neighbours, pictured in figure 1*b* (so a walker is more likely to pass between them than if they had many connections). However, it is not a global measure of closeness because the transition probability incorporates only the nearest neighbour connections between nodes (so there is no proximity between disconnected nodes, even if multiple paths exist between them). The PageRank matrix [18] *B*_*i* → *j*_ = γ*P*_*i* → *j*_ + (1 − γ)/*N* with γ a teleportation parameter gives a modified estimate of proximity, a uniform measure of closeness for disconnected nodes independent of the network's geometry.

The more refined non-backtracking matrix [36–38], as the name suggests, captures the transition probability between pairs of nodes with the walker forbidden to retrace the previous step in the reverse direction. The non-backtracking matrix has previously been used to identify a measure of centrality that does not suffer from localization for highly connected nodes [36]. A simple measure of node proximity can be established using the non-backtracking matrix, the probability of a non-backtracking walker moving between pairs of nodes in two steps. Note that in every random-walk-based case, these measures of proximity satisfy the expectations in figure 1*b* (many unshared neighbours reduce Δ_*ij*_) but not figure 1*a* (many shared neighbours increases Δ_*ij*_): a walker on blue moves to red in two steps with 50% (100%) probability using the random walk transition matrix (non-backtracking transition matrix) regardless of the number of shared neighbours. It is useful to develop a measure of closeness that incorporates these two (sometimes seemingly contradictory) aspects depicted in figure 1: nodes are close to one another if there are many paths between them, but popular nodes are less close to their neighbours than unpopular nodes.

### 2.3. The GENs: measuring closeness via a weighted harmonic mean

We have recently shown [23] that the *E*_*ij*_ or GENs, describing the topological closeness from node *j* to node *i*, satisfy the expected properties for the sharing of finite resources described in figure 1. The GENs on a weighted network of *N* nodes and *M* non-zero edges are defined as

2.1 $$\frac{W_{j}}{E_{ij}}=w_{ij}^{2}+\sum_{\begin{array}{c} l\neq i,\\ w_{jl}\neq 0 \end{array}}\frac{w_{jl}}{E_{il}+w_{jl}^{-1}},\;E_{ii}\equiv 0,$$

where *w*_*jl*_ = 0 if nodes *j* and *l* do not share an edge. This form is chosen such that the node *i* is as close as possible to itself and that if *j* is connected to only one node *k*, *j*'s closeness to *i* satisfies *E*_*ij*_ ≡ *E*_*ik*_ + *w*^−1^_*jk*_. If there are multiple paths between nodes, the closeness from *j* to *i* is strengthened if there is a direct connection between them but also includes a contribution from all other neighbours of *j* weighted by their connection strength. By choosing a harmonic mean for the form of the contribution, we bias our measure of closeness towards neighbours that themselves are close to *i*. There is no possibility of zero-valued *E*_*ij*_ for *i*≠*j* due to the offset *w*^−1^_*ij*_, avoiding the possibility of a numerical instability [39] due to a vanishing denominator. *E*_*ij*_ is thus always smaller for directly connected than indirectly connected nodes, as the contribution from direct connections in equation (2.1) is *w*^2^_*ij*_, strictly greater than *w*_*il*_/(*E*_*il*_ + *w*^−1^_*jl*_) for indirect connections. The GENs are defined using the global topology of the network, and *E*_*ij*_ is finite even for nodes *i* and *j* in the same component that share no neighbours (as may not be the case for more local measures of closeness [22]).

In appendix A, we demonstrate a number of features of the GENs when applied to synthetic networks. For homogeneous networks such as the Erdős–Rényi (ER), whose degree distribution is sharply peaked about the mean, the topological closeness between connected nodes is likewise peaked about the mean which is proportional to the mean degree of the nodes 〈*k*〉, while the closeness between disconnected nodes is dominated by the network size *N*. Networks with heterogeneous topologies, such as the Barabási–Albert networks that have a degree distribution of *P*(*k*) ∼ *k*^−3^, likewise have a scale-free distribution of the GENs for connected nodes, indicating that the GENs are indeed able to distinguish between distinct network topologies.

The nonlinear form of equation (2.1) makes analytical work intractable in all but the simplest cases, and we must generally resort to numerical work to determine the topological closeness between nodes in a network. *E*_*ij*_ can be computed numerically in an iterative fashion [23], with *E*_*ij*_ ≡ *E*^(∞)^_*ij*_ and the recursive definition $W_{j}/E_{ij}^{\left(t+1\right)}=\sum_{l}w_{jl}/[E_{il}^{\left(t\right)}+w_{jl}^{-1}]$ (with the constraint that *E*^(*t*)^_*ii*_ = 0 continually enforced). In this paper, the iteration is halted when $\max_{ij}|E_{ij}^{\left(t+1\right)}-E_{ij}^{\left(t\right)}|\leq \epsilon =0.005$. The method also requires an initial guess, *E*^(0)^_*ij*_, with *E*^(0)^_*ij*_ = 1 used in this paper.

The iterative method for evaluating equation (2.1) to determine the closeness of all nodes towards a particular node *i* requires $\sum_{\;j\neq i}k_{\;j}=M-k_{i}$ evaluations (one for each neighbour of *j*). As there are *N* target nodes, a complete evaluation of the GENs requires *O*(*NM*) computations, at worst *O*(*N*^3^) for dense networks. This scaling is problematic for large dense networks, but the worst-case scaling of *N*^3^ is common for many existing measures of centrality [15]. We note that other pairwise measures of proximity (such as resistance distance or MFPT) will generally require a matrix inversion, at a typical cost of *O*(*N*^3^) and thus comparable to the cost of evaluating the GENs. We also note that the evaluation of the set {*E*_1*,j*_} is independent of the evaluation of {*E*_2,*j*_}, meaning the calculation of the GENs can be parallelized to provide a significant boost in the speed of evaluation.

In addition to other existing measures of proximity that satisfy the expectations of figure 1, there is a great deal of functional freedom in writing equation (2.1). For example, any measure $E_{ij}^{\left(g\right)}$ of the form $W_{\;j}g(E_{ij}^{\left(g\right)})=w_{il}^{2}+\sum_{l\neq i}w_{\;jl}g(E_{il}^{\left(g\right)}+w_{lk}^{-1})$ will satisfy the desired behaviour depicted in figure 1 for a monotonically decreasing *g*(*x*), with *g*(*x*) = *x*^−1^ in the definition of equation (2.1). Another alternative definition replaces the direct weight between adjacent nodes, *w*^−1^_*lj*_, with the closeness, *E*_*lj*_, in the denominator of equation (2.1): $W_{\;j}/{\tilde{E}}_{ij}=w_{ij}^{2}+\sum_{l\neq i}w_{\;jl}/({\tilde{E}}_{il}+{\tilde{E}}_{lj})$ (with the constraint *E*_*ii*_ = 0 and *E*_*ij*_ > 0 imposed). While these alternative definitions may be of interest in certain contexts, we continue to use equation (2.1) throughout this paper, due to its simplicity and previously demonstrated successes in prediction algorithms [23] and community detection methods [11]. Variations in the definition of *E*_*ij*_ will certainly change the numerical values of the closeness, but the qualitative behaviour of the closeness between nodes is expected to be robust to perturbations of the definition of the GENs.

## 3. Centrality and topological closeness

### 3.1. Erdős centrality and mean importance

The GENs incorporate a simple idea of what is meant by the ‘closeness’ between nodes in a network where limited resources are shared, and we expect that a node *j* that is topologically close to node *i* (having small *E*_*ij*_) considers node *i* to be ‘important’ in some sense. We may therefore regard the inverse of the closeness between nodes (*ψ*_*ij*_ = *E*^−1^_*ij*_) as an unnormalized personalized measure of importance, allowing a ranking of all nodes in the network from the perspective of the node *j*. Because *ψ*_*ij*_ measures the importance of *i* from a particular node *j* (rather than the network at large), it is not equivalent to a centrality measure.

Having defined a pairwise measure of the importance a node *j* assigns to *i* using *ψ*_*ij*_, we naturally expect that we can leverage this definition into a global measure of the importance of node *i*. There already exists a wide variety of methods for measuring centrality from a global perspective, including the degree [15,40,41], PageRank [18,41], random walk [13], betweenness [13,15] and non-backtracking [36] centralities. Each measure tends to rank high-degree nodes above low-degree nodes in complex networks, but take the global network topology into account in different ways. The importance of global topology is perhaps most clear in betweenness centrality, where high-degree nodes often have high centrality, but nodes of low degree that act as bridges between components of the network may have high centrality.

**Figure 2.**
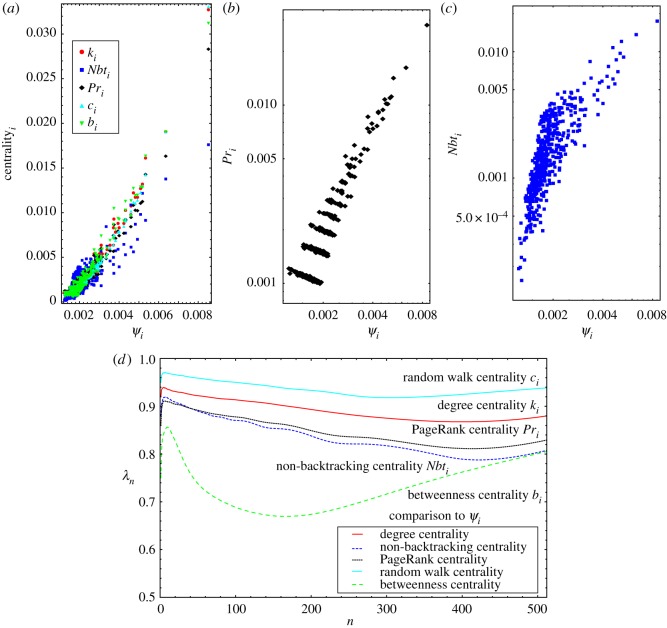
Centrality for a Barabási–Albert network with 〈*k*〉 = 20. (*a*) The Erdős centrality (*x*-axis) compared to the five common centrality measures (*y*-axis) shows an obvious positive correlation overall. Circles shows degree centrality, squares PageRank, diamonds betweenness centrality, up-triangles random walk centrality and down-triangles non-backtracking centrality. (*b*,*c*) Betweenness centrality and PageRank compared to Erdős centrality on logarithmic axes, showing the clustering due to degree in one case (*b*, betweenness) but not the other (*c*, PageRank). (*d*) The intersection metric λ_*XY*_(*n*) is used to quantify the similarity between the top *n* elements of the Erdős centrality (**o**_E_(*n*)) and the top *n* elements of the other centrality measures for varying *n*.

To convert our personalized importance measures into a single global measure for an unweighted network, we define $\Psi_{i}=\sum_{l\in C_{i}}\psi_{il}$ as the sum of the importance the neighbours of *i* assign to it (akin to the approach of [32]), which we refer to as an Erdős centrality. In figure 2*a*, we compare *Ψ*_*i*_ to a variety of other measures of centrality for a single realization of a Barabási–Albert network [7] (generated using the algorithm described in appendix B) with *N* = 512 and 〈*k*〉 = 4. In all cases, there is correlation between these various measures but with differences between the numerical values of the centrality measures for both central and non-central nodes alike. The clear correlation seen here is consistent with other realizations of the BA network, other values of 〈*k*〉, and is also seen in ER networks (not shown).

Figure 2*b*,*c* shows the same data plotted logarithmically for PageRank (*b*) and the non-backtracking (*c*) centralities in comparison with *Ψ*_*i*_ for one realization of the network. The degree of each node can contribute significantly to its centrality depending on the measure, and the clustering of the data in figure 2*b* is driven by nodes with identical degree with different nearby network topologies that lead to differing values for the GENs. Non-backtracking centrality is less dependent on node degree (as evidenced by the lack of clustering), indicating the other topological features of the network are important using this measure.

The clustering of some measures of centrality tends to occur for predominantly low-degree (and thus low-centrality) nodes, and it is preferable [20,42] to focus our comparison of the different measures on high-degree nodes [19,20]. We compare the Erdős centrality ordering to the other measures of centrality using the fractional intersection between the top-*n* orderings [43], $\lambda_{XY}(n)=(1/n)\sum_{k=1}^{n}|o_{X}(k)\cap o_{Y}(k)|/k$, with **o**_*X*_(*k*) the top-*k* ordering using method *X*. In figure 2*d*, λ_*XY*_(*k*) is plotted for *X* = *Ψ*_*i*_ and *X* the other centrality measures, averaged over 100 realizations of the network. We see that comparison of other measures of centrality to the Erdős centrality exhibits a high degree of overlap at *n* = 1 with a sharp jump in λ for *n*≲10 in all measures. Beyond *n* ≳10, there is a slow variation, but all top-*n* lists remain similar above 80–90% with the exception of the non-backtracking centrality. Despite their different formulations, the top-*n* list for *Ψ*_*i*_ compares best to the list from random walk centrality (dashed turquoise line) above 90% for low- and high-degree nodes, indicating *Ψ*_*i*_ is most closely related to the random walk centrality over all node degrees.

### 3.2. Importance eigenvector centrality and teleportation in random walks

The Erdős centrality, $\Psi_{i}=\sum_{l\in C_{i}}\psi_{il}$ described in the previous section, is a natural definition arising from the pairwise importance *ψ*_*ij*_ assigned to it by all of its direct neighbours. While well correlated with other centrality measures (suggesting its utility), a significant amount of information regarding the global importance is neglected: the value of the importance assigned to nodes that are not directly connected to *i* are all ignored. This is true of many centrality measures, generally counting the number of direct paths between nodes to identify an overall measure of importance (degree, random walk and betweenness all proceed solely through direct links between nodes).

PageRank centrality differs from a purely random-walk-based measure by accounting for indirect links between nodes through the steady state probability of a Markov process with transition probability **B**_*ij*_ = γ*a*_*ij*_/*k*_*i*_ + (1 − γ)/*N*. In this process, the random walker moves between connected nodes (randomly) with probability γ, but jumps between disconnected nodes (again, randomly) with probability (1 − γ). The leading eigenvector of the matrix **B** reduces to solving the coupled equations $Pr_{i}=N^{-1}(1-\gamma )+\gamma \sum_{\;j\in C_{i}}k_{\;j}^{-1}Pr_{\;j}$ with *C*_*i*_ the set of nodes connected to *i* (in a directed network, this is the set of nodes with edges directed towards *i*).

In the limit of γ = 0, *Pr*_*i*_ = *N*^−1^ is uniform as is expected for pure teleportation. In the limit of γ = 1 (no teleportation), the PageRank equation reduces to $Pr_{i}=\sum_{\;j\in C_{i}}k_{\;j}^{-1}Pr_{j}$, and it is straightforward to see that the anzatz *Pr*_*i*_ = *k*_*i*_/*N* is a solution (as the equation becomes $Pr_{i}=\alpha k_{i}=\alpha \sum_{\;j\in C_{i}}1$). A uniform probability of teleporting between distant nodes may be an imperfect model for the dynamics of a random walker on a network and a number of modifications to the PageRank algorithm have been proposed that account for inhomogeneous teleportation probabilities between nodes [44,45] in a variety of contexts.

A similar Markov process strongly related to the PageRank algorithm can be defined using personalized importance: a random walk performed with a transition probability $B_{\;ji}^{\prime }=\psi_{ij}/\sum_{l\neq i}\psi_{lj}$ (with the convention **B**′_*ii*_ = 0, meaning the walker never remains at *i*). This process has an interpretation similar to that of PageRank: the most probable transition for a walker at node *j* to make passes through direct connections (moving to *i* with *w*_*ij*_ > 0), but has a non-zero probability of jumping to a disconnected node. Unlike the PageRank methodology, a walker in this process has a non-uniform probability of choosing to move along an edge versus teleportation.

As an example of the heterogeneity of the teleportation in this process, a node *i* with degree *k* = 1 in an unweighted network will have a most probable transition to its sole neighbour (with the greatest importance *j* assigns going to *i* with *ψ*_*ij*_ = 1). However, the total probability of teleporting (moving from *i* to a node without a direct connection) is $p_{\mathrm{teleport}}\equiv \sum_{\;j\neq C_{i}}p_{i\to j}=1-{\left(\sum_{l\neq i}\psi_{li}\right)}^{-1}$. In appendix A, we show that the average closeness felt between disconnected nodes in a large network scales as *E*_*d*_ ∼ *N*^1/2^, which suggests that $(\sum_{l\neq i}\psi_{li})∼N^{-1/2}$. This indicates that walkers at low-degree nodes will usually teleport to more important nodes in the network (as $p_{\mathrm{teleport}}∼1-1/\sqrt{N}\approx 1$ for large *N*). Teleportation between distant nodes in the network will be highly heterogeneous in this walk, and we expect it to have a significant contribution to the centrality for large networks with low-degree nodes.

**Figure 3.**
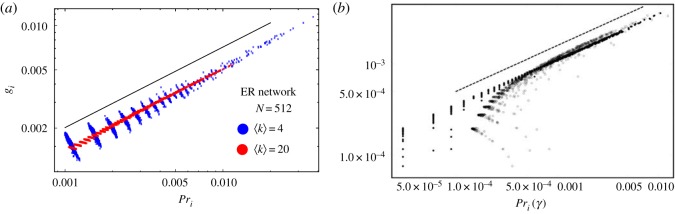
Importance eigenvector centrality *g*_*i*_ extracted from the transition matrix defined by pairwise importance **B**′. (*a*) Shown are 10 realizations of BA networks with *N* = 512 nodes: 〈*k*〉 = 20 (red) and 〈*k*〉 = 4 (blue). An approximate scaling of *g*_*i*_ ∝ *k*^*α*_*g*_^_*i*_ is observed, with the best fit of *α*_*g*_ = 0.55 for the different ensembles. The behaviour of ER networks is similar, but with greater clustering of the observed PageRank values (not shown). (*b*) Comparison of the importance eigenvector centrality *g*_*i*_ with PageRank at γ = 1 (filled circles, pure random walk) and γ = 0.85 (empty circles, 15% teleportation probability) for the largest connected component of the political blogs network [46]. The dashed line shows a scaling of *g*^2^_*i*_ ≈ *Pr*_*i*_. Disagreement between the two methods in PageRank's teleportation parameter primarily effects the ordering of low-degree nodes, which become more homogeneous for increasing γ.

The leading eigenvector of the matrix **B**′ can be compared to that of the PageRank transition probability matrix **B**, which has a uniform probability of teleporting to any node in the network (regardless of the network topology). In figure 3*a*, we show the steady-state probability of being found at a node *i* for this random walker in this process, computed from the leading eigenvector of **B**′ with elements *g*_*i*_, termed importance eigenvector centrality in this paper. A clear correlation with the degree centrality is observed, with the solid line indicating a scaling of *g*_*i*_ ∝ *k*_*i*_^*α*_*g*_^ for *α*_*g*_ ≈ 0.55. A similar quality of fit is found for larger *N* (discussed further below) as well as for the ER networks (not shown). Excellent agreement is found for high-degree nodes (as was the case in §3.1 for the Erdős centrality), with deviations occurring primarily for low-degree nodes that are clustered based on the node's degree. For all nodes of a fixed degree *k*, PageRank will tend to give a higher centrality to those nodes that are connected to high-degree hubs. By contrast, importance eigenvalue centrality *g*_*i*_ will tend to give a lower centrality as the hub's attention is divided among many nodes and it assigns a lower importance to its neighbours. This effect produces the downward slope in the clusters of data in figure 3*a*, and is more pronounced for low-degree nodes.

The relationship between PageRank and the importance eigenvector centrality *g*_*i*_ persists even for real-world networks with neither a homogeneous nor scale-free degree distribution, such as the lognormally distributed 2004 political blogs network [46]. In this network, each node is a liberal or conservative blog in the lead-up to the 2004 presidential election and each edge indicates a link between the blogs. In order to implement the GENs in equation (2.1) on this network, we converted the network from a directed network (where *w*_*ij*_≠*w*_*ji*_) to an undirected network (where *w*_*ij*_ = max(*w*_*ij*_, *w*_*ji*_)) and retained only the largest connected component of 1222 nodes. In figure 3*b*, we see *g*^2^_*i*_ and *Pr*_*i*_ are both highly correlated with the degree centrality (*R*^2^ = 0.999 and 0.982, respectively), indicating that both measures are dominated by node degree rather than other details of the network topology (as was the case in the BA networks in figure 3*a*). In the case of PageRank, this is due to the fact that hubs are connected to low-degree nodes, so walkers on low-degree nodes tend to move towards high-degree nodes if they do not teleport (occurring 85% of the time). In the case of importance eigenvector centrality, the model is entirely different: with more than 90% probability walkers on low-degree nodes (*k* ≲ 10) will teleport, but preferentially teleport to high-degree nodes. Despite the different dynamics in the walks, the steady-state probability of arriving at any node is nearly identical in both cases.

## 4. Understanding dynamics on networks through topological closeness

### 4.1. SIR model on an ER network

**Figure 4.**
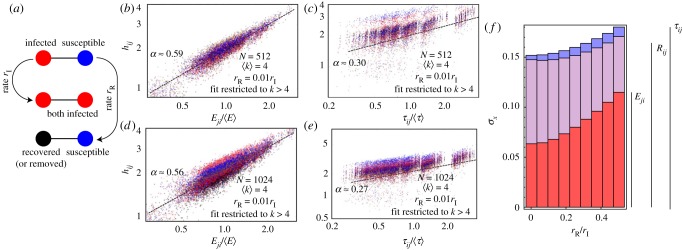
The harmonic mean of the infection time of node *j* with a single initially infected node *i*, *h*_*ij*_ in an ER network. The SIR model is diagrammed in (*a*). (*b*–*e*) compare *h*_*ij*_ with the GENs *E*_*ji*_ (*b*,*d*) for *N* = 512 and 1024 respectively on log–log axes, and the MFPT *τ*_*ij*_ (*c*,*e*) for *N* = 512 and 1024, restricted to nodes with *k*_*j*_ > 4 in all cases. The *x*-axes are scaled by the mean to permit comparison, and do not affect the scaling. Different colours denote different values of *i*, and the dashed lines denote the best fit of *h*_*ij*_∝*x*^*α*_*x*_^_*ij*_ for *x*_*ij*_ = *E*_*ji*_ or *τ*_*ij*_, respectively. The variations in *τ*_*ij*_ in *c* and *e* relative to the best fit are significantly larger than for *E*_*ji*_ in *b* and *d*. (*f*) The standard deviation of the residuals various fits (with a lower value indicating a stronger relationship between *x*_*ij*_ and *h*_*ij*_) as a function of the recovery rate for *N* = 512 and 〈*k*〉 = 4. (*f*) shows the deviation for high-degree nodes *k*_*j*_ > 4 (the behaviour is shown in appendix C for all *k*_*j*_), with lower values of *σ*_*x*_ indicating better agreement between the observed infection times and the best fits based on the measure of closeness *x*.

The spreading of an epidemic has been studied by many authors and in a wide range of contexts [16,17,47–49], with the susceptible-infected-recovered (SIR) model being one of the simplest and most commonly used models. The SIR model assumes that a population of susceptible individuals becomes infected due to interactions with previously infected individuals, and infected individuals may recover and become non-infectious. A simple schematic of the SIR model is shown in figure 4*a*, with infections occurring at a constant rate, *r*_I_, due to direct interactions between individuals, and the recovery at constant rate, *r*_R_. A number of more complex models have been considered extensively for a homogeneously mixed population of individuals [49], but non-uniform interactions between individuals, represented by networks, can have a profound impact on the dynamics of epidemic spreading in the SIR model [4,16,17]. The existence of epidemic thresholds [4,50] for homogeneous networks (or the lack thereof for scale-free networks [16]) are well-studied global quantities of interest [51], while more local quantities such as the probability of a particular node *i* becoming infected, sparking an epidemic [52], and quarantine or immunization strategies [48,53] have also been examined.

While it is clearly useful to understand the global properties of the epidemic (such as the expected number of infected individuals), a particular individual *j* may also be interested in its own probability of becoming infected given the origin of the disease and may reasonably be less concerned if no neighbours are infected than if many neighbours are infected. However, it is not straightforward to analytically calculate how long the disease will take to reach *j* from any point in the network, and it would be useful to have a measure for how ‘close’ the epidemic is from an individual node. If the infection begins with a single node *i*, we expect that the disease will more rapidly propagate to nodes for which *i* is topologically close, and it is therefore worthwhile to compare the pairwise infection times (infection time of node *j* given an initial infection at *i*) with measures of topological closeness, such as the resistance distance *R*_*ij*_, MFPT in a random walk *τ*_*ij*_, and the GENS *E*_*ij*_. PageRank and betweenness are single-node properties (not properties of a pair) and cannot be used for comparison. The resistance distance and MFPT in a random walk can be computed directly from the graph Laplacian **L** [14,15].

To see the relationship between infection time and topological closeness, we simulate an SIR epidemic (diagrammed in figure 4*a*), using Gillespie dynamics [54] on an ER graph (with a uniform probability of connection and each node having 〈*k*〉 = 4 or 〈*k*〉 = 20) and *N* = 512. The infection rate *r*_I_ = 1 and recovery rate are varied, but always above the epidemic threshold [4,16] *r*_I_ > *r*_R_/〈*k*〉. Even above the epidemic threshold, the disease may stochastically die off, and we take the pairwise infection time to be the harmonic mean of the infection time of a node *j* given an initial infection at *i* over all of the simulations, $h_{ij}^{-1}=\sum_{k=1}^{K}{\left[t_{i\to j}^{\left(k\right)}\right]}^{-1}$ with *K*_*i*_ simulations initiated at site *i* for each *r*_R_. To compute the infection time *h*_*ij*_ between all nodes, *K*_*i*_ = 100 simulations were run for every node *i* being the sole infected node at *t* = 0.

### 4.2. Comparing topological closeness with infection time

The infection time can be compared to a variety of measures of topological closeness, and in this section we focus on the GENs (*E*_*ji*_), the MFPT in a random walk (*τ*_*ij*_) and the resistance distance (*R*_*ij*_). Infection that originates at a high-degree node (*i*) will rapidly spread throughout the network, but infections starting at a low-degree node will tend to spread only locally until a high-degree node is encountered. We thus expect the rate of infection of a non-nearest neighbour (*j*) of the initial infection site *i* to be positively correlated with its topological closeness using all three measures.

In figure 4*b*–*e*, we compare *h*_*ij*_ in a network with *N* = 512 and 〈*k*〉 = 4 to *E*_*ji*_ (*b*, *d*) and *τ*_*ij*_ (*c*, *e*), normalized by $\langle E\rangle =N^{-2}\sum_{ij}E_{ij}$ (since the GENs do not contain any dynamic information and the numerical values are thus arbitrary) and $\langle \tau \rangle =N^{-2}\sum_{ij}\tau_{ij}$ (for comparison with the GENs), respectively. The figures show a random sample of 20 target nodes *j* with *k*_*j*_ > 4 (for which there is a consistent relationship for 〈*k*〉 = 4, discussed further in appendix C). As expected, infection times of non-nearest neighbours are lowest for nodes that are topologically close (low *E*_*ij*_ or *τ*_*ij*_), with the lines showing an empirical power-law fitting of *h*_*ij*_ ∝ *x*^*α*_*x*_^_*ij*_ for *x* = *E* or *τ*. The exponent is non-universal, depending on *N*, 〈*k*〉 and the recovery rate. It is apparent that the fit using the GENs is more robust than the MFPT, due to the clustering of *τ* (akin to the degree-driven clustering in figure 2*b*) with larger variation in *h*_*ij*_ for a given value of *τ*_*ij*_ than is seen for *E*_*ji*_. This is driven by the fact that *τ*_*ij*_ is much more strongly correlated with the degree of the target node *j* than is *h*_*ij*_ (shown in appendix C). The comparison of *h*_*ij*_ with *R*_*ij*_ has a trend similar to *τ*_*ij*_, and is not shown in the figure.

The quality of the fit between the infection time *h*_*ij*_ and any of the measures of closeness *x*_*ij*_ are shown in figure 4*f* using the standard deviation of the residuals $\sigma_{x}^{2}=N^{-1}\sum_{i}{\left(h_{ij}-cx_{ij}^{\alpha }\right)}^{2}$ for the power law best fit *h*_*ij*_ = *cx*^*α*^_*ij*_. The mean of the residuals $m=N^{-1}=\sum_{i}(h_{ij}-cx_{ij}^{\alpha })$ generally satisfies |*m*| ≲ 10^−3^ for all measures at all *r*_R_. Figure 4*f* shows that all closeness measures perform worse when *r*_R_ increases, due to the fact that node recovery is independent of the network topology. The figure also clearly demonstrates that the GENs are a significantly better predictor of the infection time than either the MFPT or resistance for spreading on an ER network, indicating that they correspond to a relevant measure of topological closeness that has an impact on the spreading process. For an ER network with 〈*k*〉 = 20, all nodes have degree *k* > 4 with high probability, and in this case the results are consistent with those pictured in figure 4*b*–*f* without restriction on the degree. For 〈*k*〉 = 20, we find that *σ*_*x*_ increases overall for each measure of proximity (all on the order of *σ*_*x*_ ≈ 0.3 − 0.4 for *r*_R_/*r*_I_ ≈ 0), as shown in appendix C. Consistent with the behaviour in figure 4, *σ*_E_ is lower than *σ*_*τ*_ and *σ*_R_ for non-zero *r*_R_/*r*_I_, indicating that the GENs remain a better predictor overall than resistance distance or MFPT.

### 4.3. Random walks and the GENs

**Figure 5.**
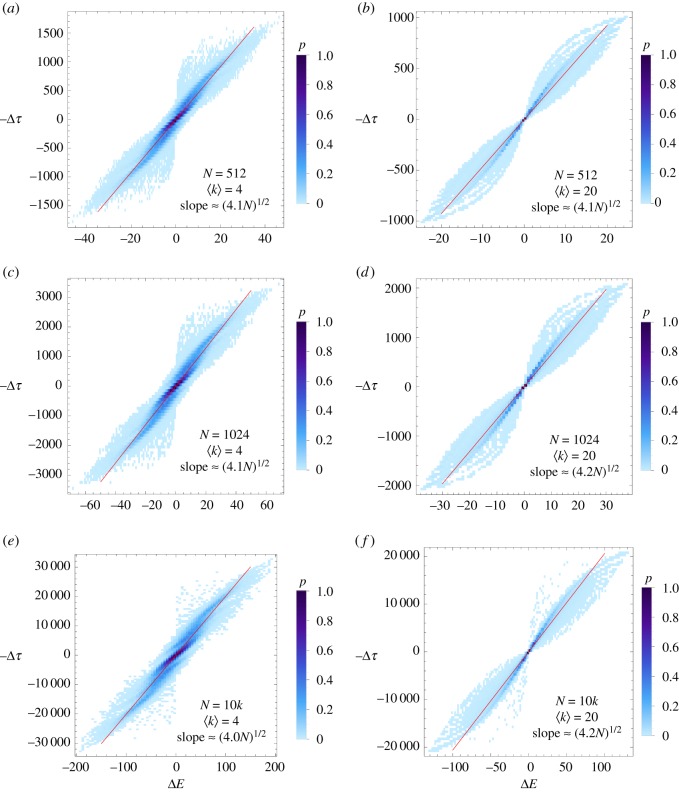
Asymmetry in the Erdős–Rényi GENs Δ*E*_*ij*_ = *E*_*ij*_ − *E*_*ji*_ compared with the asymmetry in the MFPTs for those networks, Δ*τ*_*ij*_ = *τ*_*ji*_ − *τ*_*ij*_. The colours indicate the probability *p* of seeing that value of the Δ*E* − Δ*τ* pair (counts normalized by the total number of pairs in the simulated networks). In these density plots, darker colours correspond to a greater observed frequency of the same (Δ*E*, Δ*τ*) pair. Shown are two values of *N* = 512, 1024 and 10 000 nodes as well as two values of 〈*k*〉 = 4 and 20, as indicated in the figure. The asymmetry of the GENs is highly correlated to that in the MFPT (with the slope of the best fit line indicated).

A surprising feature of figure 4 is the significant difference between the accuracy of *E*_*ji*_ and *τ*_*ij*_ in predicting the infection time. Based on the good agreement between the importance centrality *Ψ*_*i*_ and random walk centrality *c*_*i*_ in figure 2*d*, one might have expected to find consistency between the GENs and the MFPT in a random walk. Random walk centrality is defined based on the differences in MFPT [13], with *τ*_*ij*_ − *τ*_*ji*_ = *c*_*j*_ − *c*_*i*_, rather than the particular values of *τ*_*ij*_ themselves. The MFPTs are asymmetric (*τ*_*ij*_ > *τ*_*ji*_ if *i* is more easily reached than *j*), as it is easier to reach a high-degree node than a low-degree node, with a similar behaviour for the GENs (with *E*_*ji*_ > *E*_*ij*_ if *i* is topologically closer to *j* than *j* is to *i*). This suggests a comparison of the asymmetry between the two measures that could explain their agreement in figure 2*d*. In figure 5, we compare Δ*E*_*ij*_ = *E*_*ij*_ − *E*_*ji*_ to the difference in the MFPT between nodes Δ*τ*_*ij*_ = *τ*_*ij*_ − *τ*_*ji*_ for an ER network with various *N* and 〈*k*〉. The asymmetry in the MFPT is highly correlated with the asymmetry in the GENs, with an empirical scaling of $\Delta \tau_{ij}\approx -\Delta E_{\;ji}\sqrt{\alpha N}$ and *α* ≈ 4 (determined using Mathematica's FindFit function). The fact that Δ*τ*_*ij*_ ∝ Δ*E*_*ji*_ (even when there are no direct connections between *i* and *j*) again indicates that the GENs are able to capture the importance of the global network topology even for distant nodes.

## 5. Conclusion

In this paper, we have shown the utility of the GENs in measuring a non-metric topological closeness between nodes in complex networks lacking a well-defined distance metric. Derived from simple principles based on a conceptual picture of nodes sharing finite resources, the GENs incorporate the global topology of the network into a pairwise measure of closeness for connected and disconnected nodes alike. Other non-local pairwise measures can be found in the literature (e.g. the MFPT in a random walk or resistance distance between nodes), and we have shown that the GENs are able to describe the structure of and dynamics on networks in a manner consistent with or outperforming these existing measures.

The utility of the GENs was first demonstrated by identifying two potential measures of centrality derived from the GENs that identify important nodes in heterogeneous networks consistent with existing methods. The Erdős centrality, $\Psi_{i}=\sum_{l\in C_{i}}\psi_{il}$ (with *ψ*_*il*_ = *E*^−1^_*il*_), defines centrality in terms of the importance assigned by nearest neighbours and is appropriate for unweighted networks. An alternative measure of centrality that takes the importance assigned between all node pairs *i* and *j* into account arose from a novel definition of a random walk with teleportation: the importance eigenvector centrality was defined as the steady state probability of being found in a node *i* in a walk with transition probabilities *p*_*j* → *i*_ ∝ *E*^−1^_*ij*_. This is conceptually related to the teleportation probability in PageRank, but with our eigenvector centrality having an inhomogeneous teleportation probability depending on the importance of each node. In both cases, we showed that these centrality measures are consistent with existing approaches despite the very different origins they all have.

The GENs were further shown to be useful in quantifying the impact of the network topology on the dynamics on epidemic spreading on an ER network. Nodes that are disconnected but topologically close in a network should more quickly spread the infection between each other than nodes that are distant. While the resistance distance and MFPT in a random walk are both positively correlated with infection time (as expected), the GENs are an overall better predictor for high-degree nodes. We note that the dynamics of the SIR model were not chosen to match the dynamics of the epidemic spreading, as the SIR model does not have a finite resource shared between nodes (as each node can infect all of its neighbours with equal rate). The GENs are expected to perform well on predicting the infection risk of nodes for other disease models in which the process of infecting one node may reduce the infection rate of other neighbours. Taken together, the quality of the centrality measures and the correlation with dynamical processes on networks suggest that the GENs are a meaningful measure of topological proximity and may be of potential benefit in a variety of contexts.

## Figures and Tables

**Figure 6.**
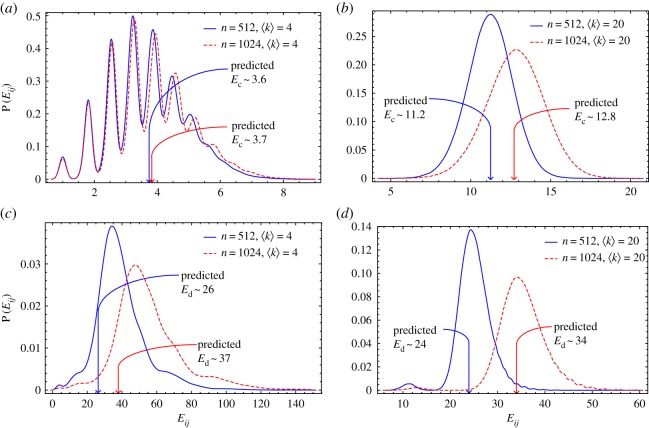
The distribution of *E*_*ij*_ split into cases where *i* and *j* are directly connected in (*a*,*b*) and not directly connected in (*c*,*d*) for ER networks with *N* = 512 and 1024 and with 〈*k*〉 = 4 (*a*, *c*) or 〈*k*〉 = 20 (*b*,*d*). Note the changing axes in all figures. The predicted average of *E*_*c*_ (equation (A 5)) and *E*_*d*_ (equation (A 4)) are marked using the same colour schemes as in the figures. For 〈*k*〉 = 20, there is excellent agreement between the theoretical and simulated means. For 〈*k*〉 = 4, the GENs are far more heterogeneous due to the larger relative fluctuations than can be captured using the simple model in equations (A 4) and (A 5), and the theoretical predictions do not agree well with the observed behaviour for both connected and disconnected nodes.

**Figure 7.**
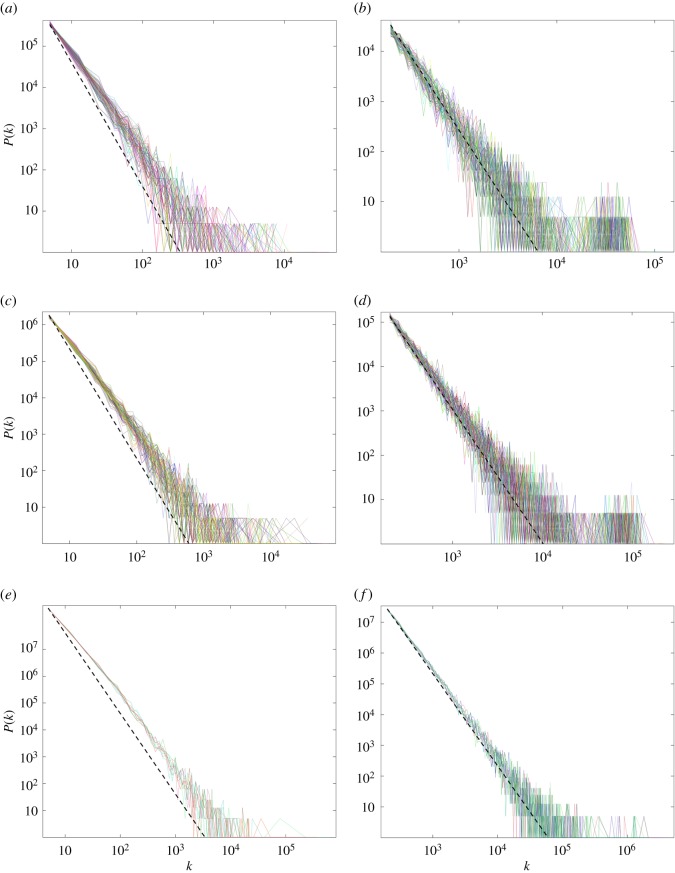
Log–log degree distributions for scale-free networks with prescribed 〈*k*〉. *P*(*k*) ∝ *k*^−3^ shown as dashed lines. Deviations from the *k*^−3^ scaling are observed for 〈*k*〉 = 4 for small *k*. (*a*) *N* = 512, 〈*k*〉 = 4 (100 networks), (*b*) *N* = 512, 〈*k*〉 = 20 (100 networks), (*c*) *N* = 1024, 〈*k*〉 = 4 (100 networks), (*d*) *N* = 1024, 〈*k*〉 = 20 (100 networks), (*e*) *N* = 10 000, 〈*k*〉 = 4 (10 networks) and (*f*) *N* = 10 000, 〈*k*〉 = 20 (10 networks).

**Figure 8.**
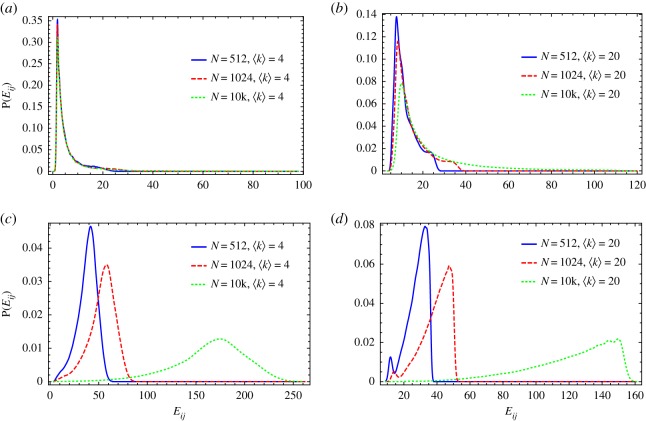
Distributions of the GENs for the Barabási–Albert networks for nodes that do share a direct connection (*a*,*b*) and do not share a connection (*c*,*d*) for 〈*k*〉 = 4 (*a*,*c*) and 〈*k*〉 = 20 (*b*,*d*) and with *N* = 512 (blue solid lines), 1024 (red dashed lines) or 10^4^ (green dotted lines). The behaviour is consistent with the ER networks in figure 6: the mean values depend strongly on 〈*k*〉 and weakly on *N* if the nodes share a direct connection, while the opposite is true if the nodes are not neighbours. Because the heavy tail of the degree distribution (with *P*(*k*) ∝ *k*^−3^) provides a broader distribution to the connected GENs distribution than in the ER case (figure 6), the mean values are further from the peaks of the distribution and are not indicated in the figure.

**Figure 9.**
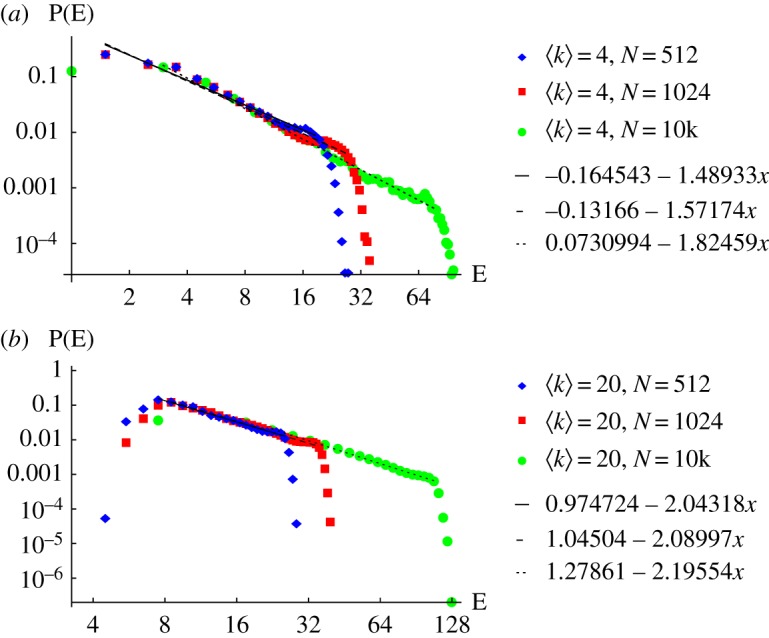
Fitting the heavy tail of the distribution of the nearest-neighbour GENs for the Barabási–Albert networks in figure 8*a*,*b* on log–log axes. Over a wide range of values, there is an approximate power-law decay which is very weakly dependent on *N* (*N* = 512 in the blue diamonds, *N* = 1024 in the red squares, and *N* = 10^4^ in the green circles) but does depend on the average connectivity (〈*k*〉 = 4 in (*a*) and 〈*k*〉 = 20 in (*b*)). This is consistent with the weak *N* dependence seen for the ER networks in equations (A 4) and (A 5).

**Figure 10.**
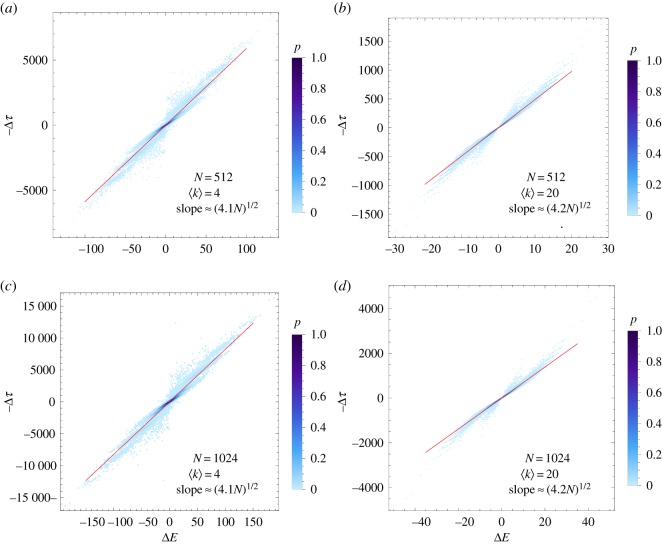
Asymmetry in the Barabási–Albert GENs Δ*E*_*ij*_ = *E*_*ij*_ − *E*_*ji*_ compared with the asymmetry in the MFPTs for those networks, Δ*τ*_*ij*_ = *τ*_*ij*_ − *τ*_*ji*_. The figure labels are identical to those in figure 5, with a similar behaviour in scaling and variation from the best fit line.

**Figure 11.**
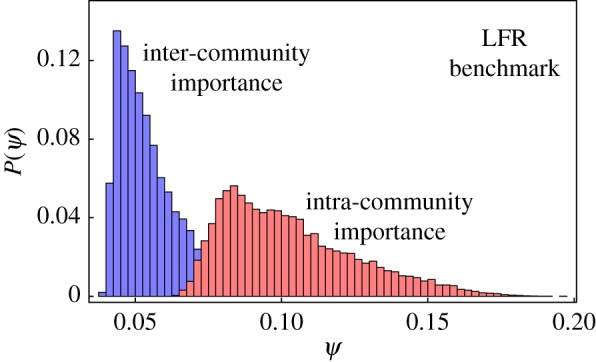
The GENs applied to the Liancichinetti, Fortunato and Radicchi benchmark [10]. The red shows the distribution of importance for nodes *i* and *j* that are in the same community but do not share a direct connection. The blue shows the distribution for those in different communities (and still sharing no direct connection). Owing to the high density of links inside of the communities, the GENs accurately indicate that *ψ*_*ij*_ is likely to be larger if *i* and *j* are in the same community.

**Figure 12.**
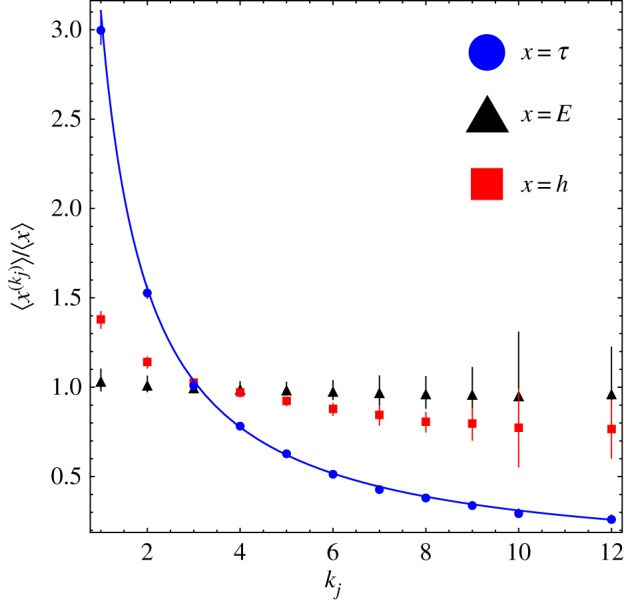
The dependence of the MFPT *τ*_*ij*_ (blue circles), GENs *E*_*ji*_ (black triangles) and mean infection time *h*_*ij*_ (red squares) as a function of the degree of a target node *j*. The MFPT is well fit by a *τ*_*ij*_ ∝ *k*^−1^_*j*_, while *h*_*ij*_ and *E*_*ji*_ have a much weaker dependence on the target node's degree. All constrained values 〈*x*^(*k*)^〉 are scaled by 〈*x*〉 (the unconstrained average over all nodes) to permit comparison.

**Figure 13.**
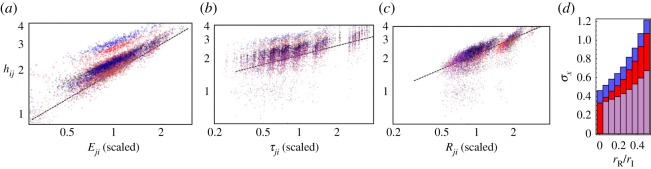
Inclusion of low-degree nodes in the prediction of *h*_*ij*_ in the fitting reduces the quality of the agreement for all measures of topological closeness for *N* = 512 and 〈*k*〉 = 4. In (*a*), low-degree nodes tend to become infected slower than would be predicted by the GENs, leading to significant weight far from the fit. (*b*,*c*) The quality of the fit for the MFPT and resistance distance (respectively). In (*d*), the poorness of the fit is quantified using the standard deviation. At *r*_R_ → 0 the GENs perform best, but resistance distance is a better predictor of infection time for larger *r*_R_. Note the change in scale from figure 4*f*.

## Data accessibility

Code to generate the GENs on any network is provided under a creative commons license for commercial and noncommercial use via [55].

## Appendix A. Distribution of the GENs in synthetic networks

**A.1. Homogeneous networks of small diameter**

While equation (2.1) is not exactly solvable for all but the simplest of network topologies, the general properties of the GENs can be explored for sufficiently homogeneous networks. The unweighted ER networks have a degree distribution sharply peaked about the mean (*k*_*i*_ ≈ 〈*k*〉, where *k*_*i*_ is the degree of the node *i* in an unweighted network), and we expect the closeness between nodes will still be broadly distributed due to the complex network topology. The mean closeness between nodes can be derived by assuming that *E*_*ij*_ = *E*_*c*_ (the ‘typical connected’ closeness) if *i* and *j* are connected, and the ‘typical disconnected’ closeness, *E*_*ij*_ = *E*_*d*_, if they are not directly connected. In an unweighted regular network, with all nodes having the same degree *k*_*i*_ = *k*, it is possible to examine the mean closeness between connected and disconnected nodes using the GENs. For homogeneous degree distributions such as the ER networks, we expect an approximation *k*_*i*_ ≈ 〈*k*〉 to be reasonable, with fluctuations in the degree expected to have a relatively minor impact, particularly for high mean degree. For these homogeneous networks, we assume that nodes that are directly connected have a typical closeness *E*_*c*_ between each other, and another closeness *E*_*d*_ ≥ *E*_*c*_ to nodes that are not. If *i* and *j* are directly connected, they have on average (*k* − 1)^2^/(*N* − 2) neighbours in common (since both have exactly *k* edges, one of which connects to the other), and they have *k*^2^/(*N* − 2) neighbours in common on average if they are not connected. A mean field approximation will treat connected (disconnected) nodes as having a fixed closeness *E*_*c*_ (*E*_*d*_) between each other, and split the sum in equation (2.1) into two parts: a sum over nodes neighbouring both *i* and *j*, and a sum over nodes only connected to *j*. This gives the approximate equations for an unweighted network of constant degree

A 1 $$\frac{k}{E_{c}}\approx 1+\frac{{\left(k-1\right)}^{2}}{N-2}\frac{1}{E_{c+1}}+\left(k-1-\frac{{\left(k-1\right)}^{2}}{N-2}\right)\frac{1}{E_{d+1}}$$

and

A 2 $$\frac{k}{E_{d}}\approx \frac{k^{2}}{N-2}\frac{1}{E_{c+1}}+\left(k-\frac{k^{2}}{N-2}\right)\frac{1}{E_{d}+1}.$$

It is possible to solve *E*_*c*_ exactly in terms of *k*, *N* and the unknown *E*_*d*_, with

A 3 $$E_{c}=\frac{2+kE_{d}^{2}-N}{-2+kE_{d}+N}.$$

Substitution of equation (A 3) into equation (A 1) and collecting terms implies that *k*^2^*E*^4^_*d*_ − *k*[*N*(*k* + 1) − 3]*E*^2^_*d*_ − 2*k*^2^(*N* − 2)*E*_*d*_ = (*N* − 2)(*k* − 1)^2^. An exact solution to this is not enlightening, but in the limit of *N* → ∞ an asymptotic solution can be found. *E*_*d*_ cannot be independent of *N* in the limit of *N* → ∞ else *E*_*d*_ would be imaginary. Rather, *E*_*d*_ must be an increasing function of *N*, implying that the highest order terms must have the same scaling, with *E*^4^_*d*_ ∼ *NE*^2^_*d*_ for large *N*. Then we expect *E*_*d*_ ∼ *N*^1/2^ to leading order, and we find for large *N* that

A 4 $$E_{d}\approx {\left(\frac{(k+1)N}{k}\right)}^{1/2}+\frac{k}{k+1}+O(N^{-1/2}).$$

Comparing this expression to the numerical solution of the equation shows less than 1% deviation for *N* ≳1000 and *k* ≲ 300, suggesting the truncation to terms of order *O*(*N*^0^) is sufficient for large *N* over a wide range of *k*. A good approximation for *E*_*c*_ can be found by setting *k* = *κN* and taking the limit of *κ* → 0. We find

A 5 $$E_{c}\approx \frac{k+2\sqrt{k^{3}/N(k+1)}}{1+\sqrt{k(k+1)/N}}\approx k+O(k^{2}N^{-1/2}),$$

where the latter is the scaling for sufficiently large *N* ≫ *k*^4^. Note that this scaling does not emerge immediately: even for *N* ≳10^4^, higher order terms can contribute in the series for only moderate values of *k*, and the full expression is required to obtain an accurate estimate for finite size networks. In an alternative limit of *N* → ∞ but *κ* = *k*/*N* finite (i.e. a large, densely connected ER network), we find the connected GENs scale as $E_{c}∼\sqrt{N}-1/\kappa +O(N^{-1/2})$, converging on the disconnected nodes $E_{d}∼\sqrt{N}+1$ but remaining closer to zero. This scaling is consistent with that for a fully connected network, with [23] $E_{c}=\sqrt{N+1}$, indicating (unsurprisingly) that a dense random network is structurally similar to a fully connected one.

**A.2. Large diameter networks**

This simple two-state approximation in equations (A 1) and (A 2) assumes there all nodes not directly connected to *i* are identical, a reasonable assumption only in the case of networks with a very small diameter. As ER networks have diameter [56] *D* ≈ log(*N*)/log(*k*) for the networks with 〈*k*〉 = 20, to a good approximation each disconnected node is only a distance 2 away from *i* for the networks considered in figure 2*b*,*d*. The approximation in equations (A 1) and (A 2) is poorly satisfied for 〈*k*〉 = 4, where the diameter is larger and fluctuations in the degree of each node are of much greater importance due to the smaller mean degree. This heterogeneity in disconnected nodes may be important for networks with small 〈*k*〉/*N* due to the larger diameter, and in the same spirit as equations (A 1) and (A 2) we define *e*_*x*_ to be the mean value of the GENs from a node *j* a distance *x* from node *i* (so *e*_1_ ≈ *E*_*c*_ in equation (A 5)). For 〈*k*〉 ≪ *N*, we can write approximately

A 6 $$\frac{k}{e_{x}}\approx \frac{1}{e_{x-1}+1}+\frac{\left(k-1\right)}{n_{x-1}+n_{x}+n_{x+1}}\left[\frac{n_{x-1}}{e_{x-1}+1}+\frac{n_{x}}{e_{x}+1}+\frac{n_{x+1}}{e_{x+1}+1}\right]$$

with *e*_0_ ≡ 0 and where *n*_*x*_ is the average number of nodes a distance *x* from node *i*. The first term accounts for the fact that a node distance *x* from *i* must be connected to at least one node distance *x* − 1 from *i*, by definition, and the second term accounts for the other potential connections: those a distance *x* − 1, *x* or *x* + 1 from *i*. Note that these are the only possible connections for a node a distance *x* from *i*, which can be connected to (a) more than one node a distance *x* − 1 from *i*, (b) other nodes a distance *x* from *i*, or (c) any number of nodes a distance *x* + 1 from *i*. In the limit of large *N* for small *k*/*N*, *n*_*x*_ ≈ *N*[1 − (1 − *k*/*N*)^*n*_*x*−1_^] ≈ *n*_*x*−1_*k*, implying that *n*_*x*_ ≈ *k*^*x*^ for connected or disconnected nodes with sufficiently small *k*/*N*. Substitution of *n*_*x*_ = *k*^*x*^ into equation (A 6) and taking the anzatz *e*_*x*_ ∼ *e*^(*λx*)^ readily shows that λ ∼ log(*k*) for sufficiently large *k* (still constraining *k*/*N* ≪ 1). The GENs thus grow exponentially for small *x*, a scaling similar to that of the GENs on a tree [23].

We empirically find that for sufficiently large *x* the growth of the GENs saturates for sufficiently large *x* (as was observed in tree networks of finite size [23]), no longer satisfying the exponential growth of *e*_*x*_ ∼ *k*^*x*^. For nodes at the diameter of the network (*x* = *D*) with *n*_*D*+1_≡0, equation (A 6) implies that *e*_*D*_ ≈ *e*_*D*−1_ + (*k* + 1)/2 + *O*(*e*^−1^_*D*−1_), taking the limit of *e*_*D*−1_ ≫ 1. In order to determine the behaviour of the GENs for a pair of nodes separated by *x* = *D* − *l* for some *l* ≪ *D*, we take the anzatz that *e*_*D*−*l*_ ≈ *e*_*D*−*l*+1_ + *ξ*_*l*_ for *ξ*_*l*_, the difference between *e*_*D*−*l*_ and *e*_*D*−*l*+1_ a function of *l* assumed small relative to *e*_*D*−*l*_. Substituting into equation (A 6) and in the limit of large *e*_*D*−*l*−1_, we find the asymptotic relationship *ξ*_*l*_ = *ξ*_*l*+1_[*k*(*k* − 1)/(*k* + 2)] + [*k* − 1 + 3/(2 + *k*)] + *O*(*e*^−1^_*D*−*l*−1_). For large *k* (but still satisfying *k*≪*N*), this implies *ξ*_*l*_ ≈ *k*(*x*_*l*−1_ + 1), and with *ξ*_0_ ≈ *k*/2 we find *ξ*_*l*_ ≈ *k*^*l*+1^/2. Asymptotically then, *e*_*x*_ ≈ *e*_*x*−1_ + *k*^*D*−*x*+1^/2 for *x* sufficiently close to *D*. The exponential growth for small *x* is therefore converted to a saturation when *k*^*x*^ ∼ *k*^*D*−*x*+1^ or when *x* ≈ (*D* + 1)/2.

For large *N* and assuming log(*k*)≫1 while still satisfying *k*≪*N*, a continuum approximation for the mean value of the disconnected GENs is determined by dividing the predicted GENs into exponential growth for *d*≲*D*/2 and a constant term for *d* ≳*D*/2. We estimate $\langle E_{d}\rangle \approx [\int_{0}^{D/2}dl\;n_{l}e^{\lambda l}+e^{\lambda D/2}\int_{D/2}^{D}dl\;n_{l}]/\int_{0}^{D}dl\;n_{l}\approx \sqrt{N}$, where *D* = log(*N*)/log(*k*) is the expected graph diameter for an ER network, *n*_*l*_ ≈ *e*^*kl*^ is the approximate number of nodes a distance *l* from *i*, and λ ≈ log(*k*) is the asymptotic growth rate of the GENs before saturation. This leads to a scaling law of $\langle E_{d}\rangle ∼\sqrt{N}$ in agreement with scaling for the two-state results, even in the limit of large *D*.

Equations (A 1) and (A 2) approximate the mean of the nonlinear terms by the function evaluated at the mean: 〈*E*^−1^〉 ≈ 〈*E*〉^−1^. Noting that $N^{-1}\sum_{l}f(x_{l})\approx f[N^{-1}\sum_{l}f(x_{l})]+\frac{1}{2}f^{\prime \prime }(\langle x\rangle )\sigma_{x}^{2}$ for any sequence {*x*_*l*_} with small variance *σ*_*x*_, we expect that the approximation underlying equations (A 1) and (A 2) tends to overestimate the value of the mean of 〈*E*^−1^〉 = 〈*E*〉^−1^ + *σ*^2^_E_/〈*E*〉^3^ ≥ 〈*E*〉^−1^ and thus our predicted value of 〈*E*_*c*_〉 is expected to be underestimates (with a similar argument true for *E*_*d*_). We emphasize here these limits are valid only for 1 ≪ 〈*k*〉 ≪ *N*, and these simplified models cannot accurately capture the statistics of low-degree networks for which the neighbour statistics cannot be captured by a simple mean value.

**A.3. Simulated distributions of the GENs for ER networks**

In figure 6, we show the distribution of the GENs for ER networks with varying *N* = 512 and 1024 and with 〈*k*〉 = 4 and 20. In figure 6*a,b* we see that changing 〈*k*〉 radically alters the mean values of *E*_*ij*_ as well as the shape of the distributions, while changing *N* only marginally affects the distribution of the connected GENs, shifting the peak a small amount while retaining a similar functional form. For 〈*k*〉 = 4 the distribution of *E*_*ij*_ exhibits multiple peaks in figure 6*a*, with each local maximum corresponding to a different degree of the node *j* and with the width of the distribution about the peak coming from differing degrees of the node *i*. Such heterogeneity is less apparent for high-degree nodes (figure 6*b*), where fluctuations in the degree of *i* or *j* have less of an impact on the GENs, and the distributions are unimodal. For disconnected nodes, the distributions have a single dominant peak (figure 6*c*,*d*), and the location of the peaks is well predicted by equations (A 4) and (A 5) for 〈*k*〉 = 20. Owing to the significance of degree fluctuations for the smaller 〈*k*〉 = 4, there are large differences between the predicted and observed means.

The growth in the mean value for the disconnected GENs for increasing *N* is due to the increasing sparsity of the network. Each node still has 〈*k*〉 neighbours on average, but a pair of nodes has only ≈〈*k*〉^2^/*N* neighbours in common for large *N*. As the size of the network increases, there will be fewer shared neighbours and the nodes will tend to be less close to one another. This has a marginal effect on the closeness between nodes that share a direct link (for which we expect *E*_*c*_ ∼ *k* for large *N*), but have a significant effect on disconnected nodes (for which $E_{d}∼\sqrt{N}$). In the limit as *N* → ∞ and for fixed *k*, an ER network will drop below the percolation threshold (with 〈*k*〉/*N* < 1) and become a set of small components; in this limit the approximations underlying equations (A 1) and (A 2) break down. For a fixed attachment probability 〈*k*〉 = *pN* with *N* → ∞, we expect the homogeneity conditions required for equations (A 1) and (A 2) to remain valid, and thus that $E_{c}∼\sqrt{N}$.

## Appendix B. GENs in heterogeneous networks

**B.1. Generation of Barabási–Albert networks**

The Barabási–Albert model generates a scale-free random network by combining the notion of growth and preferential attachment. Beginning with some small initial network (a kernel), the method works by adding new nodes incrementally, attaching each new node to existing nodes in the networks. Attachment to existing nodes is preferential in that a new node has a probability of being attached to an existing node proportional to the degree of the existing node: existing nodes with higher degree will tend to increase degree, while existing nodes with lower degree will only rarely acquire a new connection. The parameters in the model are

— *n*: number of nodes in initial clique
— *m*: number of edges in initial clique
— $k_{\min}$: degree of new node upon addition (number of new edges added at each step)
— *N*: total number of nodes
— *M*: total number of edges

and the mean degree 〈*k*〉 of a node in the final network is given by 〈*k*〉 = 2*M*/*N*. To generate a network with a prescribed 〈*k*〉, we need to choose *n*, *m* and $k_{\min}$ properly. If we require that our initial clique is fully connected, preventing any initial node from being preferred over any other at first attachment, then *m* = (*n*^2^ − *n*)/2. We can also observe that for *N*/*n* ≫ 1, $M\approx Nk_{\min}$, as each new node introduces *k* edges by definition. It is thus natural to choose this limiting case as a constraint to enforce for any network size, meaning that we require $M=Nk_{\min}$ and thus $\langle k\rangle =2k_{\min}$. This determines *m* and $k_{\min}$ and allows us to solve for initial clique size *n* by equating the total number of edges in the final network to the sum of the initial number of edges and the number of edges added by growth.

$$\begin{array}{rl} M & =m+(N-n)k_{\min},\\ Nk_{\min} & =\frac{n^{2}-n}{2}+Nk_{\min}-nk_{\min}\\ \text{and}\; & =2k_{\min}+1. \end{array}$$

So the algorithm to generate a random Barabási–Albert network can be sketched as follows. Beginning with a fully connected clique of $n=2k_{\min}+1$ nodes, add *N* − *n* new nodes incrementally. Each new node is attached to the existing nodes by choosing $k_{\min}$ unique existing nodes, each chosen with probability proportional to the existing node's degree, and add edges between the new node and this existing set to the network. Because this algorithm requires beginning with a relatively large initial clique to satisfy the mean degree constraint exactly, the final degree distributions feature heavier tails than typical scale-free graphs, especially for large values of 〈*k*〉/*N* (figure 7).

**B.2. Topological closeness in scale-free networks**

In contrast to the homogeneous degree distribution of the ER random network model, Barabási–Albert (BA) networks [7] have a scale-free, heterogeneous degree distribution, and figure 8 shows that the distribution for the GENs for BA networks are likewise heterogeneous for directly connected nodes. The distribution for the GENs between nodes that share an edge (shown in figure 8*a*,*b*) appear to have a heavy tail and approximately satisfy *Pr*(*E*_*ij*_ = *E*) ∼ *E*^−λ^ for nodes that share a direct connection, with an empirically determined scaling exponent near 1.5 for 〈*k*〉 = 4 and around 2.1–2.2 for 〈*k*〉 = 20 (shown in figure 9, found using Mathematica's LinearModelFit function). This is in comparison to the heavy tailed degree distribution with the *P*(*k*) ∝ *k*^−3^ scaling of the BA networks for both values of 〈*k*〉. Variations in the scaling exponent for *E*_*ij*_ despite the fixed scaling exponent in the degree distribution does not indicate a lack of robustness of the model: as *N* increases, each node with degree *k* > 1 is connected to a greater number of nodes with degree *k* = 1, thus decreasing the impact of shared neighbours for each node in the network. The eventual scaling of the GENs for BA networks in the limit of *N* → ∞ is not readily derived analytically, due to the heterogeneity of the networks that prevent mean field approximations as in equations (A 1) and (A 2) from being appropriate. Low-degree nodes are often linked to high-degree hubs in the BA algorithm, which leads to a significant decrease in the most probable value of *E*_*ij*_ seen in figure 9*a*,*b* compared to figure 6*a*,*b*. This is because randomly selected nodes in the homogeneous ER networks probably have degree *k*, whereas a randomly selected node *j* will most likely be of low degree in a BA network, and will have a smaller value of *E*_*ij*_ to a hub (*i*).

Interestingly, the distribution of the GENs for disconnected nodes does not depend as strongly on the scale-free nature of the degree distribution, with similar qualitative features found in both figure 6*c*,*d* for the ER networks and figure 8*c*,*d* for the BA networks. While the existence of hubs in the BA networks tends to give a higher probability of finding smaller values of *E*_*ij*_ for disconnected nodes in comparison to ER networks, the most likely values of *E*_*ij*_ are similar for disconnected nodes in either network topology (in contrast to the radically different distributions for connected nodes). We have considered only unweighted networks in this analysis, and allowing weighted edges further complicates the analysis of the ‘typical’ GEN between nodes unless a homogeneity assumption on the distribution of weights is likewise made.

Deviations from the best fit power law in figure 9 occur for large *E* due to the finite size of the network. The scale-free nature of the network does not alter the arguments used to show the saturation of the GENs for nodes at the diameter, and we therefore expect some upper bound on the maximum value of closeness. We expect an exponential growth in the GENs for nodes that are a large distance away from one another (as the network becomes more tree-like, with a low probability of overlap in the neighbours) as was seen for the more homogeneous ER networks. There also appears a lower bound on the GENs in figure 9, due to the fact that even neighbours shared between nodes reduce the closeness between them. If we imagine that two nodes with degree *k* have a direct connection between them and all neighbours are shared, representing the topology producing the lowest closeness between the pair, the GENs will be $E_{\min}∼\sqrt{k}$. This produces the lower bound at *E* ≈ 2 in figure 9*a* for 〈*k*〉 = 4 and at *E* ≈ 4.47 in figure 9*b* for 〈*k*〉 = 20.

**B.3. Asymmetry in random walks in Barabási–Albert networks**

In the main text, we found that the asymmetry in the MFPT in a random walk on an ER network was highly correlated with the asymmetry in the GENs, with a proportionality constant $\approx \sqrt{4N}$ for a wide range of *N*. In figure 10, we see a similar scaling holds for random walks on BA networks, consistent with the good agreement between the Erdős centrality and the random walk centrality for BA networks in figure 2.

**B.4. GENs in networks with community structure**

The usefulness of the nonlinear importance *ψ*_*ij*_ = *E*^−1^_*ij*_ on a network can rapidly determine meaningful relationships between nodes in complex networks. To illustrate this, we consider the benchmark of Liancichinetti, Fortunato and Radicchi (LFR) [10], which constructs a network of communities of variable sizes *n* (distributed as *P*(*n*) ∝ *n*^−*β*^), a scale-free distribution of the nodes (with *P*(*k*) ∝ *k*^−γ^), and which is characterized by the mixing parameter, *μ*, as the fraction of inter-community edges. We have previously shown [11] that the GENs can be used to detect the community structure underlying this benchmark. When measuring the importance of a node, a global measure of centrality will generally focus on nodes with high degree, but due to the heterogeneous density of edges between communities, we expect a meaningful definition of the importance *j* assigns to *i* to differ significantly depending on if *i* and *j* are in the same community.

Note that the determination of the GENs does not require or use knowledge of the community structure. In figure 11, we determine the distribution of importance *ψ*_*ij*_ between nodes *i* and *j* that do not share a direct connection (*w*_*ij*_ = 0) for nodes within *i*'s community (red) and outside of *i*'s community (blue) on an LFR network with *N* = 10^3^, *k* = 25, γ = 2, *β* = 1 and *μ* = 0.3. There is an immediately apparent difference in the distributions, with a greater probability of a high importance if *i* and *j* are in the same community due to the increased number of shared neighbours (even in the absence of a direct connection). However, the intra-community and inter-community distributions overlap, indicating that some pairs assign a greater importance across communities than another pair within the same community. This is driven by the heterogeneous node degrees, with high-degree nodes assigning little importance to any node (including within their own community) but receiving high importance from low-degree nodes (including outside of their community). Increasing the LFR parameter *μ* (which increases the number of edges between communities) reduces the difference in the distributions, but varying the other system parameters has only a minor impact on the clear distinction between the two distributions (data not shown).

## Appendix C. Topological closeness and dynamics on networks

In §4, the infection time of a target node *j* for a disease originating at node *i* was shown to be an increasing function of three different models of topological closeness: *R*_*ij*_, *τ*_*ij*_ and *E*_*ji*_. The infection time *h*_*ij*_ tended to be clustered when compared to *τ*_*ij*_ (leading to significantly greater variation in the residuals). This is driven by the very strong relationship between *τ*_*ij*_ and the degree of the target node, depicted in figure 12. To determine the relationship between degree and topological closeness, we computed $\langle \tau^{\left(k\right)}\rangle =\sum_{ij}\tau_{ij}\delta_{k,k_{j}}/\sum_{ij}\delta_{k,k_{\;j}}$ with *δ*_*x*,*y*_ = 1 if *x* = *y* and 0 otherwise. This represents the mean MFPT (averaged over all origin nodes *i* and all target nodes *j*) with the constraint that the degree of the target node is *k*. We find a very strong dependence of the MFPT on the degree of the target node, with the blue line showing *τ*^(*k*)^ ∝ *k*^−1^. This strong relationship may be unsurprising, as the steady state probability of being found at a node *j* is proportional to *k*_*j*_ (as discussed in the main text). We can likewise compute 〈*h*^(*k*)^〉 and 〈*E*^(*k*)^〉 and find they both have a much weaker dependence on the degree of the target node (error bars are standard deviation of the mean).

Figure 4 was restricted to target nodes *j* for which *k*_*j*_ > 4 for an ER network with 〈*k*〉 = 4. This is because while the infection times of low-degree nodes are still correlated with the GENs, with approximately the same exponent in the empirical fit *h*_*ij*_ ∝ *E*^*α*^_*ji*_, the value of the coefficient of proportionality *c* appears to vary with *k*_*j*_ for low-degree target nodes. This is illustrated in figure 13*a* for *r*_R_ = 0.01*r*_I_, with the dashed line the same fitting exponent as in figure 4*b* but the points corresponding to low-degree nodes (with *k*_*j*_ ≤ 4, different colours indicate different initial nodes). The best fit for the GENs tends to underestimate the infection time of low-degree nodes. The same qualitative behaviour is seen for *τ*_*ij*_ as well in figure 13*b*, with *h*_*ij*_ tending to be underestimated by the best fit. The wide variation in figure 13 is consistent with that of figure 4*c*, and we expect that *τ*_*ij*_ will be a worse predictor of the infection time than the GENs. In figure 13*c*, we see the resistance distance is qualitatively similar to the MFPT (more clustered and with greater fluctuations than the GENs), but importantly the predictions for the infection times are not systematically underestimated as they are in figure 13*a*. We find that the GENs remain a better predictor of the infection time than either *R* or *τ* for *r*_R_ ≈ 0, but that resistance distance quickly overtakes the GENs as *r*_R_ increases. It is important to note the difference in the axes between figures 13*d* and 4*f*, with the standard deviation for all three measures significantly higher with the inclusion of low-degree nodes than was seen for solely high-degree nodes.
